# Enhancing benefit finding and psychological well-being in depression care: a quasi-experimental study of group psychoeducation in Chinese adults

**DOI:** 10.3389/fpsyg.2025.1673904

**Published:** 2025-11-04

**Authors:** Xin Lu, Yu Xiao

**Affiliations:** ^1^Adult Psychiatric Male Ward, Hunan Second People’s Hospital (Hunan Brain Hospital), Changsha, Hunan, China; ^2^Department of Psychosomatic Medicine, The Second People's Hospital of Hunan Province (Hunan Brain Hospital), Changsha, Hunan, China

**Keywords:** intervention, benefit finding, depression, well-being, mental health

## Abstract

**Background:**

Major depressive disorder (MDD) is a leading cause of disability globally and is often accompanied by emotional and cognitive impairments that limit individuals’ ability to perceive positive aspects of life. Within the framework of health psychology and the biopsychosocial model, meaning-based interventions such as group psychoeducation may help enhance protective psychological resources like benefit finding and overall well-being. This study evaluated the effectiveness of a structured psychoeducational group intervention in improving benefit finding, psychological well-being, and depressive symptoms among Chinese adults with MDD in a real-world clinical context.

**Methods:**

A quasi-experimental pre-test post-test design with a control group was used. A total of 114 adults diagnosed with MDD were allocated to either an intervention groups (*n* = 57) or a control groups (*n* = 57). The intervention group participated in 8 weekly sessions, each lasting 90 min. Outcome measures included the Benefit Finding Questionnaire (BFQ), the Reiff Psychological Well-being Scale, and the Beck Depression Inventory-II (BDI-II). Data were analyzed using paired and independent t-test along with analysis of covariance (ANCOVA).

**Results:**

The intervention group showed significant increase in benefit finding, with mean scores rising from 57.12 ± 9.48 at pre-test to 83.33 ± 9.19 at post-test (*t* = 16.41, *p* < 0.001). No significant change was observed in the control group (pre-test: 56.39 ± 10.11; post-test: 56.17 ± 9.59 post-test; *t* = 0.15, *p* = 0.413). ANCOVA controlling for pre-test scores confirmed that post-test benefit finding scores were significantly higher in the intervention group compared to the control group (*F* = 28.12, *p* < 0.001) with a medium effect size (*η*^2^ = 0.31). However, there were no statistically significant improvements in psychological well-being or depression severity between groups (*p* > 0.05).

**Conclusion:**

This study supports the integration of health psychology principles into mental health care by demonstrating that group psychoeducational interventions can effectively enhance benefit finding in individuals with depression, especially in collectivistic cultural contexts. However, longer or more intensive interventions may be required to observe broader improvements in psychological well-being and symptom reduction.

## 1 Introduction

Mental disorders such as major depressive disorder (MDD) are increasingly recognized as a major global health challenge, affecting not only individuals’ emotional well-being but also their ability to function in everyday life (Vigo et al., 2016). According to the World Health Organization (2022), nearly one in eight people worldwide live with a mental health condition. Despite growing awareness, access to effective psychological care remains limited in many parts of the world, particularly in low- and middle-income countries (Esponda et al., 2020). In China, mental illness is now the leading cause of disability, yet many individuals still face barriers such as stigma, lack of awareness, and uneven access to mental health services (Ni et al., 2020). Although there have been reforms in the country’s mental health system in recent years, including the enactment of the Mental Health Law in 2013 and the expansion of social services, social stigma, lack of public awareness and unequal access to psychosocial services continue to hinder the full recovery of patients (Liang et al., 2018). Traditional treatment approaches have focused primarily on pharmacological interventions. Although these methods are essential for symptom control (Cui et al., 2024), they alone cannot address all the psychological and social aspects of the recovery process. However, increasing evidence highlights the importance of integrating psychological frameworks—particularly those rooted in health psychology—to address the broader determinants of recovery, including social, emotional, and behavioral dimensions.

In the last few decades, the importance of integrating psychoeducation and psychological interventions to enhance coping, increase self-efficacy, and promote mental health has been increasingly recognized (Sarkhel et al., 2020). One emerging psychological concept in this context is the concept of Benefit Finding, a cognitive-emotional process in which individuals identify positive changes in themselves following the experience of difficult situations, including increased resilience, personal growth, improved relationships, and greater appreciation for life (Cheng et al., 2017). Recent studies across organizational and healthcare settings have further illustrated the importance of psychosocial approaches in mental health care (Cai et al., 2024; Tuan et al., 2024; Al-Akashee et al., 2024).

Although this concept has been explored more in research related to chronic physical illnesses such as cancer (Zhu et al., 2022), heart disease (Moshki et al., 2020), and HIV/AIDS (Finkelstein-Fox et al., 2020), studies on it in the field of mental health, especially in non-Western countries, are very limited. However, some research has shown that benefit finding can act as a protective factor against depression, enhance motivation for treatment, and improve quality of life in mental patients (Chiba et al., 2020).

Importantly, this ability is not just an individual trait, but can be cultivated through structured interventions, especially in programs that focus on meaning-making, self-reflection, and social connection. Among these, group psychoeducational interventions are considered one of the most promising approaches. These interventions, which are structured, combine disease education, cognitive-behavioral skills, emotion regulation, and social support (Raya-Tena et al., 2021). Some studies have shown that these programs can increase insight into the disease, reduce relapse rates, improve coping skills, and enhance recovery (Raya-Tena et al., 2021; Martín-Carrasco et al., 2014). Furthermore, the group format of these interventions is consistent with the collectivist Chinese culture and can enhance empathy, belonging, and interpersonal learning (Wang and Liu, 2010). Despite extensive efforts in the field of psychotherapy in China, few studies have examined the effects of psycho-educational group Intervention on positive psychological changes such as benefit finding in individuals with mental disorders. Such outcomes align with the goals of health psychology, which seeks not only to reduce symptoms but also to empower individuals by fostering resilience, meaning-making, and psychological strengths within their sociocultural contexts.

In addition to finding benefit, another aspect that is important in the process of mental health recovery is psychological well-being, which includes various dimensions of an individual’s mental and emotional functioning. Psychological health includes self-acceptance, personal growth, purpose in life, independence, mastery of the environment, and positive relationships with others. These elements contribute to an individual’s overall sense of well-being and can be directly affected by interventions aimed at improving mental health. Psychoeducational group interventions can promote psychological well-being by enhancing self-awareness, increasing social support, and improving coping skills that are essential for increasing resilience and improving overall mental health. Although the role of psychological well-being in the recovery process is widely accepted, its integration into interventions, especially in non-Western cultures, remains an area that requires further investigation. In this light, psychological well-being should be considered a central outcome in health-focused psychological interventions, and integrated alongside symptom-based metrics when evaluating treatment efficacy.

Therefore, the present study seeks to fill this gap and explores the feasibility and short-term outcomes of implementing a structured psychoeducational program for individuals with MDD in China, contributing to the growing movement toward integrating health psychology principles into everyday mental health practice.

## 2 Materials and methods

### 2.1 Participants and study design

This study employed a quasi-experimental pre- and post-test control group design, conducted between June 20 and October 10, 2024, at a mental health center in Hunan, China. Eligible participants met the following inclusion criteria: diagnosis of Major Depressive Disorder (MDD) according to the Diagnostic and Statistical Manual of Mental Disorders, Fifth Edition (DSM-5), moderate depression severity based on the Beck Depression Inventory-II (BDI-II), aged between 18 and 64 years, ability to understand and speak Chinese, and willingness to participate in group sessions. Exclusion criteria included: history of other mental disorders (e.g., anxiety or bipolar disorder), the presence of chronic physical illnesses that could affect outcomes, and inability to attend the group sessions.

The minimum sample size was calculated using G*Power software (version 3.1). Based on a quasi-experimental design with two groups (intervention and control), two assessment time points (pre-test and post-test), significance level of *α* = 0.05, power of 0.80, and an expected medium effect size of 0.50 [according to Cohen’s criteria and previous studies (Cuijpers et al., 2014)], a minimum of 54 participants was required. To allow for an anticipated dropout rate of 10–20%, the sample was increased to 66 participants.

Assignment to intervention and control groups was non-random but stratified based on demographic variables (e.g., age and gender) to improve baseline comparability. Participants in both groups continued to receive their usual pharmacological and psychotherapeutic treatments prescribed by their psychiatrist, while the intervention group additionally received a structured psychoeducational group program. This non-randomized approach, coupled with real-world implementation, justifies the classification of this study as quasi-experimental rather than a randomized clinical trial.

Routine treatments in this study included medication, regular psychiatric visits, and individual counseling or brief psychotherapy, consistent with current clinical guidelines for depression treatment in China (Liu et al., 2025). In this study, pre-test data was collected before the intervention, and post-test data was collected after the 8-week intervention period.

The study was conducted in accordance with the Declaration of Helsinki and was approved by the Ethics Committee of The Second People’s Hospital of Hunan Province (NO: 2024018). All participants provided written informed consent.

### 2.2 Diagnosing major depression using DSM-5

According to the DSM-5, a diagnosis of MDD requires the presence of at least five depression symptoms during the same two-week period. These may include depressed mood, loss of interest or pleasure, sleep disturbances, weight or appetite changes, feelings of worthlessness or excessive guilt, and recurrent thoughts of death or suicide (Nussbaum, 2020). In this study, all participants were clinically assessed by a licensed psychiatrist to confirm the diagnosis and to rule out attributable mental or physical conditions.

### 2.3 Depression severity

Depression severity was assessed using The BDI-II, a well-validated self-report tool used to measure depression levels in both adults and adolescents (Wang et al., 2011). It consists 21 items, and each scored from 0 to 3, depending on symptom severity in the past 2 weeks. The total score ranges from 0 to 63, with higher scores reflecting more severe depression. Categories include: 0–13 (minimal or no depression), 14–19 (mild), 20–28 (moderate), and 29–63 (severe). To ensure a homogeneous sample, only individuals scoring between 20 and 28 (moderate) were included. The Chinese version of the BDI-II has demonstrated excellent validity and reliability in previous studies (Wang et al., 2011).

### 2.4 Benefit finding questionnaire (BFQ)

Benefit Finding was measured using the Chinese version of the Benefit Finding Questionnaire (BFQ), validated for use with mental health conditions (Cao et al., 2024). The BFQ includes 21 items covering domains such as personal growth, social relationship enhancement, resilience, acceptance, helping others, and reprioritization. Responses are given on a 5-point Likert scale (1 = strongly disagree to 5 = strongly agree). Higher total scores indicate greater benefit finding in response to life challenges (Cao et al., 2024). The BFQ has demonstrated strong construct validity and high internal consistency (Cronbach’s alpha = 0.89).

### 2.5 Psychological well-being

Psychological well-being was assessed using the 18-item Chinese version of Ryff Psychological Well-being scale, which has been culturally adapted and validated for the Chinese population (Chen et al., 2013; Li, 2014). The scale assesses six main components of psychological well-being: self-acceptance, personal growth, purpose in life, environmental mastery, autonomy, and positive relations with others. Each domain is represented by three items. Participants responded using a 6-point Likert scale (1 = strongly disagree to 6 = strongly agree), with some items reverse-scored. Higher total scores reflect greater psychological well-being.

### 2.6 Intervention

A structured group psychoeducational intervention was developed based on prior research (Raya-Tena et al., 2021; Bademli et al., 2023; Sawyer et al., 2023) and aimed to improve self-efficacy, psychological flexibility, and adaptive coping among participants with depression. The intervention consisted of eight weekly sessions, each lasting 90 min, conducted over a two-month period in a face-to-face group format. Sessions were facilitated by a licensed clinical psychologist with more than 5 years of experience in leading group-based interventions for depression. Each session focused on a specific component of psychological well-being, with clearly defined objectives, exercises, and discussion themes. The content was informed by principles from cognitive-behavioral therapy (CBT), positive psychology, and meaning-centered approaches. A brief overview of the session content is as follows: Session 1: Introduction to the intervention, overview of benefit finding, and its relevance in managing depression. Participants were encouraged to share personal experiences of hardship and explore how meaning and strength can be drawn from these experiences. Session 2: Exploration of meaning-making processes and identifying personal growth. Through guided discussions and narrative reflection, participants learned to recognize positive transformations that can arise from challenging life events. Sessions 3 and 4: Training in cognitive restructuring and thought reframing techniques. Participants practiced identifying automatic negative thoughts (ANTs) and replacing them with more balanced and constructive cognitions, using CBT worksheets and group role-playing. Sessions 5 and 6: Focused on interpersonal effectiveness and building social support networks. Topics included assertive communication, active listening, and trust-building activities. Participants engaged in partner exercises to enhance relational skills and share feedback. Sessions 7 and 8: Development of psychological resilience, setting personal goals, and planning for post-intervention application. Participants created individualized action plans to integrate the strategies into their daily routines and were encouraged to reflect on future-oriented growth.

Each session incorporated a combination of psychoeducation (10–15 min), experiential activities (20–30 min), group discussions (30 min), and reflective writing exercises (10–15 min). These components were designed to foster emotional awareness, cognitive insight, peer support, and behavioral activation. Participants were also provided with weekly handouts and homework assignments to reinforce learning between sessions. The summary of the intervention is presented in Table 1.

**Table 1:** Summary of all session’s group psychoeducational intervention.

Session	Main theme/focus	Core activities and techniques	Intended outcomes/psychological processes
1	Introduction and Benefit Finding	Overview of intervention and concept of benefit finding; sharing personal experiences of hardship; discussion of meaning and strength derived from adversity	Orientation to group process; emotional expression; initial meaning-making
2	Meaning-Making and Personal Growth	Guided discussions and narrative reflection to identify positive transformations arising from challenges	Recognition of growth and reappraisal of adversity; fostering psychological flexibility
3	Cognitive Restructuring I	Psychoeducation on automatic negative thoughts (ANTs); identifying maladaptive cognitions; group practice using CBT worksheets	Cognitive insight; increased self-awareness; challenging negative thinking
4	Cognitive Restructuring II	Continued practice with thought reframing; role-playing adaptive responses to stressful situations	Internalization of cognitive restructuring skills; strengthening adaptive coping
5	Interpersonal Effectiveness I	Training in assertive communication, active listening, and trust-building	Enhanced communication; developing social competence and connectedness
6	Interpersonal Effectiveness II	Partner exercises and feedback on relationship dynamics; building supportive networks	Reinforcement of social support; increased sense of belonging and empathy
7	Resilience and Goal Setting	Exploration of personal strengths; development of individualized action plans; identifying barriers and facilitators	Strengthening resilience; promoting goal-directed behavior and self-efficacy
8	Consolidation and Future Growth	Review of skills learned; reflection on personal progress; planning for post-intervention application and maintenance	Integration of coping strategies; empowerment; sustaining benefit finding over time

### 2.7 Statistical methods

Descriptive statistics (mean, standard deviation, percentages, and frequency) were calculated for key variables. Independent samples *t*-tests were used to compare baseline variables between the groups. Paired t-test assessed within-group changes from pre- to post-test. Additionally, analysis of covariance (ANCOVA) was conducted to assess post-test differences while controlling for baseline scores. Partial eta squared (*η*^2^) was reported as the measure of effect size for ANCOVA analyses. No formal correction for multiple comparisons was applied, as the number of primary outcomes was limited and all analyses were pre-specified and confirmatory in nature. Nonetheless, marginal *p*-values should be interpreted with caution due to the potential for inflated Type I error. Due to the low dropout rate, a per-protocol analysis was performed, focusing on participants who completed the full intervention. Participants who dropped out of the study (*n* = 7) were excluded from the final analysis. This approach was chosen to assess the effects of the intervention on those who adhered to the study protocol. All analyses were performed using SPSS version 26, with significance set at *p* < 0.05.

## 3 Results

**Figure 1:**
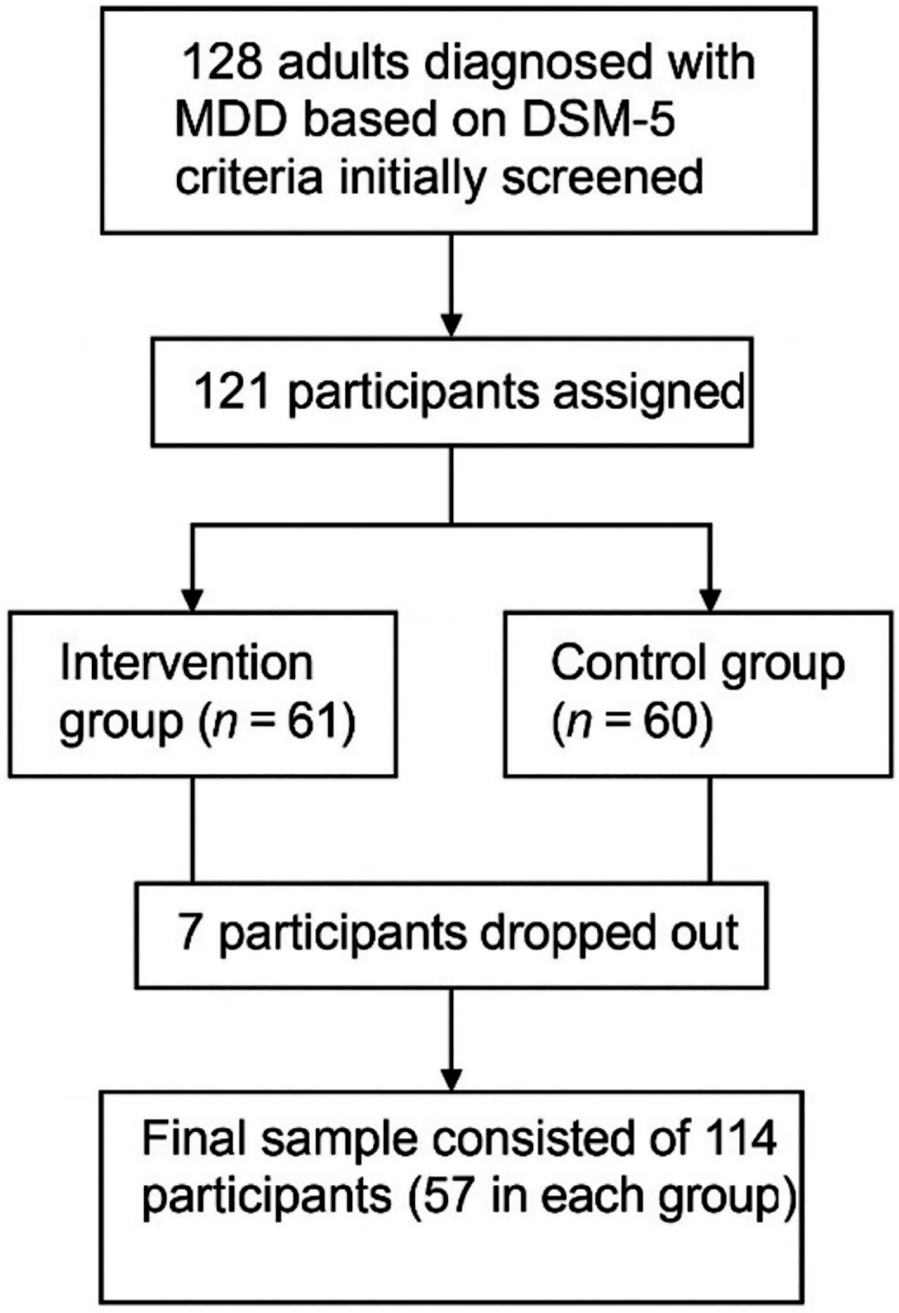
Participant recruitment and retention flowchart.

A total of 128 adults diagnosed with MDD based on DSM-5 criteria were initially screened at a mental health center in Hunan, China. Following the eligibility screening, 121 participants were assigned to either the intervention (*n* = 61) or control (*n* = 60) group. During the study, seven participants dropped out due to missing more than two consecutive sessions, unwillingness to continue, or relocation (four participants from the intervention group and three participants from the control group). The final sample consisted of 114 participants (57 in each group, age = 35.4 ± 3.2 years) (Figure 1).

Overall, 50 (43.9%) were female. Twenty participants (17.5%) had completed only secondary education and 54 participants (47.3%) were employed full-time (see Table 1).

Independent samples *t*-tests showed no statistically significant differences in age (35.6 ± 3.1 in the intervention group vs. 35.2 ± 3.3 in the control group), Benefit Finding scores (57.12 ± 9.48 in the intervention group vs. 56.39 ± 10.11 in the control group), Psychological Well-being scores (63.82 ± 8.83 in the intervention group vs. 63.67 ± 8.93 in the control group), and BDI-II score (24.31 ± 2.17 in the intervention group vs. 24.58 ± 2.12 in the control group) (*p* > 0.05). These results confirm that the groups were comparable prior to the intervention (Table 1).

Paired t-tests were used to examine changes in scores of Benefit Finding, Psychological Well-being, and Depression Severity in each group from pre-intervention to post-intervention (Table 2). In the intervention group, the Benefit Finding score increased significantly, with a mean of 57.12 (SD = 9.48) at pre-intervention to 83.33 (SD = 9.19) at post-intervention (*t* = 16.41, *p* < 0.001). Although the Psychological Well-being score also increased from 63.82 (SD = 8.83) to 67.28 (SD = 9.69), this change was not statistically significant (*t* = 1.91, *p* = 0.061). Also, the Depression Severity score in this group decreased from 24.31 (SD = 2.17) to 22.94 (SD = 2.35), but this decrease was not statistically significant (*t* = 1.93, *p* = 0.058). In the control group, no significant changes were observed in any of the variables examined (*p* > 0.05) (Table 3).

**Table 2:** Baseline demographic and psychological characteristics of participants by group.

Variable		All (*n* = 114)	Intervention group (*n* = 57)	Control group (*n* = 57)
Age (years), *M* ± SD		35.4 ± 3.2	35.6 ± 3.1	35.2 ± 3.3
Gender (female), *n* (%)		50 (43.8%)	26 (45.6%)	24 (42.1%)
Education status, *n* (%)	Secondary education	20 (17.5%)	10 (17.5%)	10 (17.5%)
	Vocational diploma or college-level education	52 (45.6%)	27 (47.4%)	25 (43.9%)
	Bachelor’s degree or higher	42 (36.9%)	20 (35.1%)	22 (38.6%)
Employment status, *n* (%)	Full-time employed	54 (47.37%)	28 (49.12%)	26 (45.61%)
	Part-time employed	27 (23.7%)	13 (22.8%)	14 (24.56%)
	Unemployed	33 (28.93%)	16 (28.08%)	17 (29.83%)
Benefit finding, *M* ± SD		56.75 ± 9.80	57.12 ± 9.48	56.39 ± 10.11
Psychological well-being, *M* ± SD		63.75 ± 8.88	63.82 ± 8.83	63.67 ± 8.93
Depression severity, *M* ± SD		24.44 ± 2.14	24.31 ± 2.17	24.58 ± 2.12

Note: *M* ± SD indicates mean ± standard deviation.

**Table 3:** Within-group comparisons of pre- and post-test scores (*M* ± SD).

Variable	Group	Pre-test	Post-test	*t*	*p*
Benefit finding	Intervention	57.12 ± 9.48	83.33 ± 9.19	16.41	<0.001
	Control	56.39 ± 10.11	56.17 ± 9.59	0.15	0.413
Psychological well-being	Intervention	63.82 ± 8.83	67.28 ± 9.69	1.91	0.061
	Control	63.67 ± 8.93	62.73 ± 9.19	−0.65	0.517
Depression severity	Intervention	24.31 ± 2.17	22.94 ± 2.35	−1.93	0.058
	Control	24.58 ± 2.12	24.22 ± 2.21	−0.97	0.336

Note: *M* ± SD indicates mean ± standard deviation.

To evaluate the effectiveness of the group psychoeducational intervention, separate analysis of covariance (ANCOVA) was performed on post-intervention scores for benefit finding, psychological well-being, and depression severity. In each analysis, pre-intervention scores were included as a covariate to control for baseline differences and isolate the true effect of the intervention. The assumption of homogeneity of regression slopes was confirmed (*p* > 0.05), validating the use of ANCOVA.

After adjusting for pre-intervention scores, the intervention group demonstrated significantly higher benefit finding compared to the control group (*F* = 28.12, *p* < 0.001). The adjusted mean was 81.8 (SE = 1.2) in the intervention group and 58.1 (SE = 1.1) in the control group, yielding a mean difference of 23.7 (95% CI: 19.2 to 28.2). The effect size was moderate (partial *η*^2^ = 0.31). Psychological well-being did not differ significantly between groups (*F* = 2.37, *p* = 0.237), with adjusted means of 65.3 (SE = 1.5) for the intervention and 63.9 (SE = 1.4) for the control group. The mean difference was 1.4 (95% CI: −0.9 to 3.7), indicating a small and non-significant effect (partial η^2^ = 0.03). Depression severity showed a non-significant reduction in the intervention group (*F* = 3.12, *p* = 0.081). Adjusted mean scores were 23.0 (SE = 0.4) for the intervention and 24.0 (SE = 0.4) for the control group, with a mean difference of −1.0 (95% CI: −2.2 to 0.2) and a small effect size (partial *η*^2^ = 0.04) (Table 4).

**Table 4:** Results of ANCOVA for post-test scores (controlling for pre-test).

Variable	Group	Adjusted mean ± SE	*F* (1,111)	*p*-value	Partial *η*^2^	Mean difference (95% CI)
Benefit finding	Intervention	81.8 ± 1.2	28.12	<0.001	0.31	23.7 (95% CI 19.2 to 28.2)
	Control	58.1 ± 1.1				
Psychological well-being	Intervention	65.3 ± 1.5	2.37	0.237	0.03	1.4 (95% CI –0.9 to 3.7)
	Control	63.9 ± 1.4				
Depression severity	Intervention	23.0 ± 0.4	3.12	0.081	0.04	−1.0 (95% CI –2.2 to 0.2)
	Control	24.0 ± 0.4				

## 4 Discussion

The present study aimed to investigate the effectiveness of a group psychoeducational intervention on Benefit Finding, Psychological Well-being, and Depression Severity in adults with MDD in China. To the best of the researchers’ knowledge, few studies have explored the role of group psychoeducational interventions in enhancing finding benefits, particularly in Chinese depressed populations. This focus aligns with the principles of health psychology, which emphasize adapting interventions to cultural and contextual realities in order to enhance mental health outcomes.

One of the key findings was a significant increase in benefit finding among participants in the intervention group. Post-intervention scores were also significantly higher than those in the control group, with a moderate effect size. These results are consistent with previous research showing that meaning-oriented or psychoeducational interventions can foster benefit finding in individuals with chronic conditions such as cancer, Alzheimer’s disease, and anxiety disorders (Cheng et al., 2012; Morgado et al., 2022). Overall, these findings indicate that group-based psychoeducation can effectively foster benefit finding among individuals with depression, highlighting its potential role in promoting positive psychological adaptation during recovery.

A possible explanatory mechanism can be drawn from the Posttraumatic Growth (PTG) theory by Tedeschi and Calhoun (2004), which posits that exposure to psychological struggle can trigger cognitive restructuring and foster a sense of growth and personal meaning. The current intervention included core elements such as experience sharing, reflective dialog, and resilience training—components that may have catalyzed such cognitive-emotional shifts. Furthermore, within the Chinese collectivist context, group-based interactions may have reinforced social connectedness and perceived support, thereby amplifying the intervention’s effects (Xie et al., 2023). While this interpretation aligns with collectivist cultural norms that value social harmony and interdependence, it remains a hypothesis rather than a confirmed explanatory mechanism. The present study did not include direct cultural or qualitative assessments—such as measures of collectivism, perceived group cohesion, or participants’ narratives about interpersonal experiences—so this explanation remains inferential. Future studies could incorporate validated scales or qualitative interviews to examine how cultural values shape engagement, mutual support, and perceived benefit in group-based interventions. Such mixed-method designs would help clarify the specific cultural processes that enhance intervention efficacy in collectivist contexts.

While collectivism may enhance social connectedness, it could also discourage open emotional expression due to concerns about maintaining harmony and avoiding burdening others. Therefore, future programs should consider culturally sensitive approaches that foster both group cohesion and individual authenticity.

Benefit finding is understood as the ability to cognitively reframe adversity and derive meaning from suffering. Key intervention mechanisms may include positive reappraisal, gratitude, and the activation of personal strengths. These elements were integral to the present intervention and are supported by Fredrickson’s Broaden-and-Build Theory of Positive *Emotions*, which suggests that positive emotions (e.g., hope, gratitude) can expand cognitive flexibility and build lasting psychological resources (Gong et al., 2018). These mechanisms reflect how emotional and cognitive flexibility—key components of psychological resilience—can be intentionally developed through structured psychosocial care, reinforcing the importance of integrating health psychology frameworks into routine depression treatment (Gong et al., 2018).

Although the significant increase in Benefit Finding scores in our intervention group aligns with theoretical frameworks such as PTG theory and the Broaden-and-Build theory, it is important to emphasize that this interpretation is cautious. In this study, we did not directly measure the process variables proposed by these theories (e.g., positive emotions or cognitive flexibility); therefore, we cannot, based on the current data, confirm any mediating role or causal relationship between the intervention and the increase in Benefit Finding. Conclusions regarding mechanisms are hypothetical and theoretically consistent, and future research should be designed to directly measure mediators (e.g., PANAS for positive emotions and a cognitive flexibility scale) in order to examine causal pathways.

Previous research has demonstrated that abbreviated psychosocial interventions, including Mindfulness-based stress reduction programs delivered over 3 to 7 weeks, can produce significant and lasting improvements in psychological outcomes (Fortney et al., 2013; Hallman et al., 2014; Virgili, 2015). These findings support the theoretical sufficiency of our eight-session group psychoeducation format in enhancing psychological well-being. Also, in current, all participants in the intervention group attended all eight sessions, which reflects excellent adherence and strengthens confidence that the intervention was delivered as intended. At the same time, this lack of variability in attendance meant that a dose–response analysis was not feasible, and thus we cannot empirically determine whether fewer or more sessions would have produced different effects. Future studies should therefore consider designs that systematically vary the number of sessions (e.g., 4, 8, or 12 weeks) and incorporate intermediate assessments to directly examine whether eight sessions are sufficient, or whether greater “dosage” yields larger or more sustained improvements in psychological well-being.

In this study, the intervention did not produce statistically significant improvements in psychological well-being or depressive symptom severity. While adjusted post-intervention scores for both variables were slightly better in the intervention group than in the control group, these differences did not reach statistical significance, and the corresponding effect sizes were small, suggesting limited clinical relevance. Several factors may explain these findings. First, Ryff’s model of psychological well-being involves multidimensional and relatively stable constructs—such as autonomy, purpose in life, and environmental mastery—that often require prolonged and deeper therapeutic engagement to shift meaningfully (Fava and Tomba, 2009; Ryff and Singer, 2008). The brevity of the current intervention, which emphasized psychoeducation and existential meaning rather than sustained behavioral or cognitive change, may not have been sufficient to produce robust improvements. This aligns with previous research indicating that brief interventions are less effective in enhancing complex psychological traits unless supplemented with longer-term therapeutic components (Ryff, 1989; Blasco-Belled and Alsinet, 2022). Taken together, the findings suggest that short-term psychoeducational programs may be insufficient to produce meaningful improvements in the deeper dimensions of psychological well-being, which likely require extended engagement and reinforcement.

Second, the non-significant change in depression severity may reflect the limited capacity of benefit-finding-focused psychoeducation to directly alleviate core depressive symptoms. Unlike interventions grounded in cognitive-behavioral therapy (CBT) or behavioral activation—which explicitly target negative thought patterns and behavioral avoidance—this intervention prioritized meaning-making and self-reflection. While potentially valuable for long-term resilience, such a focus may be insufficient for short-term symptom reduction, especially in clinical populations with moderate depression. This explanation is supported by evidence suggesting that integrated approaches—combining psychoeducation with CBT or pharmacotherapy—are more effective in reducing depressive symptoms (Sin and Lyubomirsky, 2009; Ojala et al., 2019; Brownie et al., 2023). This further supports the need for multimodal approaches that combine symptom-focused treatments with meaning-centered and resource-based interventions, a balance often advocated in integrated health psychology models. In sum, while the intervention was beneficial in promoting meaning and resilience, its limited effect on depressive symptoms underscores the importance of integrating symptom-focused components for more comprehensive treatment outcomes.

Integrating cognitive-behavioral or behavioral activation components into psychoeducational programs could strengthen both emotional and cognitive mechanisms targeted by our intervention. While the present intervention primarily emphasized meaning-making, self-reflection, and resilience, the inclusion of structured CBT elements (e.g., cognitive restructuring, identifying negative automatic thoughts) or behavioral activation techniques (e.g., scheduling rewarding or value-consistent activities) could directly target maladaptive cognitions and behavioral withdrawal—core features of depression. Evidence from hybrid interventions combining positive psychology or meaning-focused content with CBT or behavioral activation demonstrates that such integrative approaches can simultaneously enhance benefit finding, promote psychological well-being, and reduce depressive symptoms (Ameli, 2016; Kubzansky et al., 2018; Lindhe et al., 2023). Therefore, blending symptom-oriented and growth-oriented techniques may optimize both clinical and psychosocial outcomes in future applications of this model.

Although our intervention significantly enhanced benefit finding, it did not lead to reductions in depressive symptoms. This pattern suggests that the program operates more as a resource-building than a symptom-reducing intervention, primarily fostering meaning-making, resilience, and adaptive coping. Accordingly, group psychoeducation programs of this kind may not serve as stand-alone treatments for acute depression, but rather as complementary or adjunctive approaches within the broader continuum of depression care. Consistent with prior studies (Vos and Vitali, 2018; Ortega-Maldonado and Salanova, 2018; Rosa et al., 2025), the primary impact of positive psychology and meaning-focused interventions, is often observed in domains of personal growth, psychological well-being, and adaptive coping, rather than in direct symptom remission. Clinically, this implies that these interventions may be best positioned as preventive or resilience-enhancing strategies for individuals with subclinical symptoms, or as adjuncts to evidence-based treatments (e.g., CBT, pharmacotherapy) for patients with clinical depression, helping to foster long-term adaptation and improve quality of life.

This study has several limitations that should be considered in interpreting the findings. First, this study was conducted in a single mental health center in Hunan Province, China, which may limit the generalizability of the findings to other regions or broader populations. Sociocultural and institutional factors unique to this setting might influence the intervention’s outcomes. Future studies should aim to replicate the findings in diverse geographic and clinical contexts to enhance external validity. Second, all outcome measures in this study were based on self-report questionnaires, which may introduce response biases such as social desirability or recall bias. Given the group-based nature of the intervention, participants may have felt motivated to provide socially desirable responses, potentially inflating the perceived effectiveness of the program. Future research should consider incorporating clinician-rated assessments or objective behavioral measures to triangulate findings and reduce subjectivity. Future studies could also include qualitative components (e.g., interviews or participant reflections) to better understand how individuals internalize and apply benefit-finding principles, offering richer insight into the psychological processes underlying observed quantitative changes. Third, participation in this study was entirely voluntary, which introduces the possibility of selection bias. Those who chose to enroll may differ systematically in terms of motivation, psychological openness, or other unmeasured characteristics that could affect the outcomes. Also, the study did not implement evaluator blinding; outcome assessors were aware of group allocation, which may have inadvertently influenced their evaluations. Future studies should aim to blind outcome evaluators to reduce the potential for assessment bias.

Additionally, no correction for multiple comparisons was applied, which may have increased the risk of Type I error. Therefore, the observed significant results should be interpreted cautiously and viewed as preliminary until replicated in larger samples. Finally, although the sample size was adequate, future studies could benefit from larger and more diverse samples to validate and extend the findings. Furthermore, the study did not include a follow-up assessment to evaluate the durability of the intervention’s effects over time. This limits our understanding of whether the observed improvements in benefit finding are sustained beyond the immediate post-intervention period. Longitudinal designs are recommended to assess the long-term clinical impact of psychoeducational interventions. Future studies should also distinguish between transient emotional improvements and enduring psychological changes. Incorporating follow-up assessments or state–trait measures would help clarify whether benefit-finding gains reflect temporary mood shifts or lasting psychological growth.

## 5 Conclusion

This study provides preliminary evidence that group psychoeducational interventions can significantly enhance benefit finding among adults with major depressive disorder. This supports the potential value of meaning-based approaches in helping individuals reinterpret adversity and strengthen inner psychological resources. However, the limited impact on depressive symptoms and psychological well-being suggests that short-term interventions may be insufficient for complex clinical outcomes. These findings point to the need for integrated strategies that combine meaning-making with symptom-targeted care, as encouraged by the health psychology perspective. Furthermore, this study highlights the importance of culturally responsive psychological interventions in East Asian mental health contexts—programs that not only reduce symptoms but also empower individuals and promote sustainable recovery through psychosocial growth. Future research should explore how these interventions can be embedded within broader health systems to ensure accessibility, continuity, and long-term impact. Overall, this study underscores the potential of culturally grounded, meaning-oriented psychoeducational interventions to complement conventional depression care, fostering both recovery and psychological growth within collectivist contexts.

## Data Availability

The raw data supporting the conclusions of this article will be made available by the authors, without undue reservation.
